# The association of reduced left ventricular strains with increased extracellular volume and their collective impact on clinical outcomes

**DOI:** 10.1186/s12968-021-00776-7

**Published:** 2021-07-05

**Authors:** Chunna Jin, Jonathan Weber, Harsimar Singh, Kathleen Gliganic, J. Jane Cao

**Affiliations:** 1 St Francis Hospital & Heart Center, 100 Port Washington Blvd., Roslyn, NY 11576 USA; 2 State University of New York At Stony Brook, 100 Nicholls Road, Stony Brook, NY 11794 USA; 3 Department of Cardiology, Second Affiliated Hospital of Zhejiang University, Hangzhou, Zhejiang, 310009 China

**Keywords:** Extracellular volume, Cardiomyopathy, Strain, Heart failure

## Abstract

**Background:**

Myocardial fibrosis and left ventricular (LV) longitudinal strain are independently associated with adverse clinical outcomes. However, the relationship between tissue properties and strain indices as well as their collective impact on outcomes are yet to be fully elucidated. We aim to investigate the relationship between LV global longitudinal strain (GLS), global circumferential strain (GCS) and global radial strain (GRS) with extracellular volume (ECV) and their collective impact.

**Methods:**

Consecutive patients referred for clinical cardiovascular magnetic resonance (CMR) due to cardiomyopathy were prospectively enrolled. All patients underwent CMR with T1 mapping. ECV was calculated incorporating native and post-contrast T1 as well as hematocrit. LV GLS, GCS, and GRS were assessed by feature tracking. Hazard ratios and Kaplan–Meier curves were produced to assess the association between strains and T1 mapping indices with a composite outcome of all-cause mortality and hospitalized heart failure.

**Results:**

The study consisted of 259 patients with mixed referring diagnoses of non-ischemic/ischemic cardiomyopathy and 21 normal controls. Decreased GLS, GCS and GRS were associated with increased ECV, increased native T1, and reduced post-contrast T1 in a dose dependent manner when T1 or ECV was in the abnormal range. After a mean follow-up of 31 ± 23 months, 41 events occurred including 37 heart failure admissions and 4 deaths. Kaplan–Meier plots demonstrated that reduced strains were associated with reduced event-free survival predominantly in patients with increased ECV (≥ 28.3%). The worst outcome was among those with both reduced strains and increased ECV. In the multivariable models, increased ECV, reduced post-contrast T1 and reduced strains in all 3 directions remained predictors of outcome risk, respectively.

**Conclusion:**

Our findings highlight the intrinsic link between altered CMR tissue properties and impaired myocardial mechanical performance and additionally demonstrate improved risk stratification by characterizing tissue property among patients with reduced strain.

## Introduction

Increased extracellular volume fraction (ECV), assessed by cardiovascular magnetic resonance imaging (CMR), is a valuable surrogate of myocardial fibrosis burden and is associated with adverse clinical outcomes [1–3]. Left ventricular (LV) global longitudinal strain (GLS), an index of mechanical performance is also a robust predictor of outcome risk [3–5]. However, it remains unclear how tissue property alteration assessed by ECV is related to myocardial mechanical change evaluated by strain, not only in the longitudinal direction, but also in the circumferential and radial directions, in clinical cohorts with diverse etiologies and how they collectively impact adverse clinical outcomes. In this prospective study we sought to test the hypotheses that decreased strains are associated with abnormally increased ECV and collectively they are associated with greater risk of adverse outcomes.

## Methods

### Participants

This was a prospective study approved by the institutional review board. Informed consent was obtained from all participants who were recruited from patients referred for clinical CMR due to a referring diagnosis of cardiomyopathy at our institution between January 2012 and December 2018. All participants completed a questionnaire for demographic information and medical history. The clinical charts were reviewed to confirm cardiovascular history. Exclusion criteria included implanted pacemaker or defibrillator, renal insufficiency with estimated glomerular filtration rate < 45 ml/min/1.73 m^2^, significant arrhythmia, claustrophobia and metallic hazards. Normal controls were recruited among subjects without cardiovascular history or risk factors and had a normal electrocardiogram (ECG), transthoracic echocardiogram and CMR.

### CMR image acquisition

All subjects underwent scans in a 1.5 T CMR scanner (Avanto, Siemens Healthineers, Erlangen, Germany) with an 8-element phased array surface coil. No adenosine or regadenoson stress was administered. Cardiac volumes and systolic function were assessed using balanced steady-state free precession cine images with retrospective ECG gating during a breath-hold. Average temporal resolution was 50 ms. A stack of short axis planes (8 mm thickness, no gap) and 3 long axis planes (2-, 3-, and 4-chamber) were obtained using the following imaging parameter: echo time (TE) 1.3 ms, repetition time (TR) 3.1 ms, flip angle 70°, and average in-plane resolution 1.3 × 1.3 mm^2^. T1 mapping was performed with a modified Look Locker inversion recovery sequence with a 3(3)5-scheme before and 15 min after contrast administration [6]. Native and post-contrast T1 were acquired from 3 short-axis slices (basal, mid and apical). The apical slice was chosen from the most proximal slice of the apical segment to avoid partial volume. The same T1 mapping sequence was used for the duration of the study. For late gadolinium enhancement (LGE) imaging a phase sensitive inversion recovery (PSIR) gradient echo sequence was performed 8–10 min after the administration of 0.15 mmol/kg of gadopentetate dimeglumine (Magnevist, Bayer Healthcare, Berlin, Germany) following TI scout imaging to determine the optimal inversion time.

### CMR image analysis

Biventricular volumes and ejection fraction (EF) were analyzed based on a consecutive short axis stack of cine images using commercially available QMASS software (Medis Medical Imaging, Leiden, The Netherlands). LV volume and mass were normalized to body surface area.

T1 values of the myocardium and blood pool were assessed in the pre- and post-contrast T1 mapping images. We averaged T1 values of the basal, mid and apical slices. Hematocrit was drawn on the day of CMR. ECV was calculated using the formula:

$${\text{ECV}} = (\Delta {\text{R1}}_{{{\text{myocardium}}}} /\Delta {\text{R1}}_{{{\text{blood}}}} )*({\text{1 - hematocrit}}),$$

where R1 = 1/T1 time [7].

**Fig. 1:**
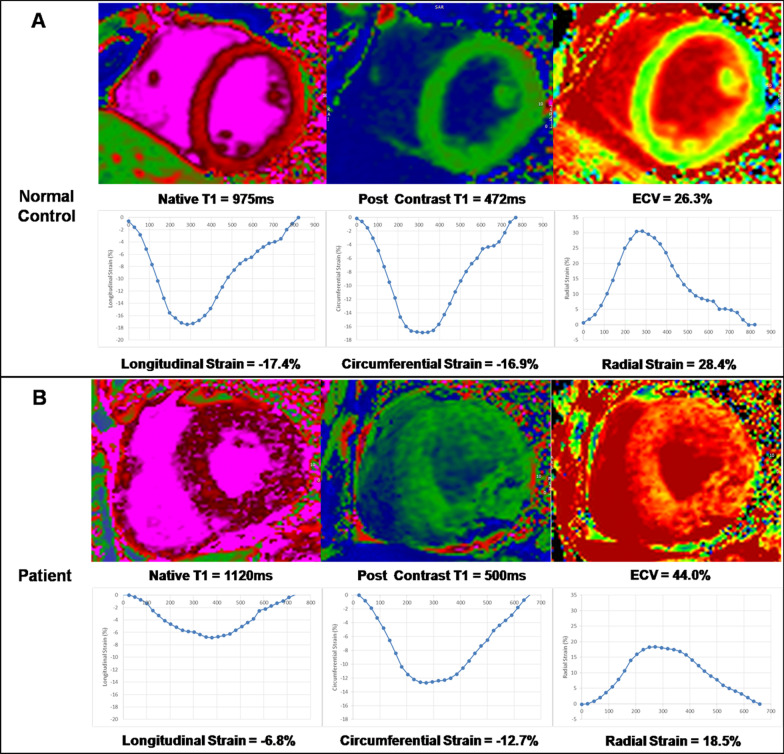
Examples of native T1, post-contrast T1 and extracellular volume (ECV) in a normal control (A) and in a patient (B) and the corresponding profiles of longitudinal, circumferential, and radial strains. **A** Upper panel shows a normal subject with normal global native T1 (975 ms), post-contrast T1 (472 ms), and ECV (26.3%); Bottom panel shows the same normal subject with normal global longitudinal strain (GLS) (− 17.4%), global circumferential strain (GCS) (− 16.9%), and global radial strain (GRS) (28.4%). **B** Upper panel shows a patient with increased global native T1 (1120 ms), increased ECV (44.0%), and preserved global post-contrast T1 (500 ms). Bottom panel shows the same patient with reduced GLS (− 6.8%), reduced GCS (− 12.7%), and relatively preserved GRS (18.5%)

LV strains, including GLS, global circumferential strain (GCS) and global radial strain (GRS) were analyzed using feature tracking on cine images. The feature tracking contours of the LV endocardium and epicardium were drawn manually at end diastole which were propagated automatically to all phases [8]. Contours were manually corrected when automatic tracking failed. The peak strain values for GLS, GCS and GRS were determined on the strain curves where GLS and GCS were of negative values and GRS was of positive value. Both T1 mapping and feature tracking analyses were performed using commercial software (cvi42, version 5.6.5, Circle Cardiovascular Imaging Inc., Calgary, Alberta, Canada). The examples of ECV and strain profiles from a normal control and a patient are shown in Fig. 1. To test the reproducibility of T1 mapping we randomly selected 15 cases and evaluated by 2 readers with consensus reading. The reproducibility of strain was reported by our group previously using the same program [9].

### Statistical analyses

Continuous variables were described as mean ± standard deviation (SD). Categorical variables were presented as frequency and percentage. Continuous variables were compared between groups using Student’s *t*-test. Categorical variables were compared using chi-square or Fisher’s exact test as appropriate. The Jonckheere–Terpstra test was applied to test the linear trend for non-parametric data. The primary outcome of interest was a composite endpoint which included all-cause mortality (taken from the National Death Index) and inpatient heart failure admissions obtained from electronic medical records in our six-hospital health care system. A receiver operating characteristic (ROC) curve analysis was performed to create optimal cut points by using the maximum of Youden’s Index (as sensitivity + specificity – 1). Unadjusted survival functions were produced using Kaplan Meier curves stratified by LV strain as well as ECV. Multivariable Cox proportional hazards models were performed to estimate the average hazard ratio (HR) and 95% confidence interval (CI) across the follow-up period. Important clinical confounders included in the models were age, gender, hypertension, diabetes and presence of an infarct pattern on LGE. An alpha of 0.05 was considered statistically significant for confidence intervals and p-values. Inter-class correlation coefficients (ICC) were calculated for reproducibility of T1 mapping. SAS  (version 9.4, SAS Institute Inc., Cary, North Carolina, USA) was used for analysis.

## Results

Our study consisted of 21 normal controls and 259 patients with mixed diagnoses including non-ischemic/ischemic cardiomyopathy. The categories of cardiomyopathy and baseline characteristics are listed in Table 1. In the patient group, the mean age was 54 ± 13 years and 196 (76%) were males. The mean LV ejection fraction (LVEF) was 51 ± 11% with 36% (N = 93) having reduced LVEF (< 50%). The prevalence of any LGE was 48% (125) among patients. All strain parameters were lower in patients than in normal controls: GLS: − 10.5 ± 3.5% vs − 13.5 ± 1.9% (p < 0.001); GCS: − 12.9 ± 4.1% vs − 16.2 ± 2.4% (p < 0.001); GRS: 19.6 ± 8.3% vs 25.9 ± 5.8 (p < 0.001). However, ECV was not significantly different between patients (27.7 ± 5.1%) and normal controls (25.9 ± 2.6%) (p = 0.11).

**Table 1:** Baseline characteristics of study population

	Diseased patients (N = 259)	Normal controls (N = 21)	P-value
*Clinical data*			
Age (years)	54 (13)	44 (15)	0.001
Male	196 (76)	13 (62)	0.163
Body surface area (m^2^)	2.06 (0.24)	1.86 (0.21)	< 0.001
Body mass index (kg/m^2^)	29.4 (5.1)	24.9 (3.2)	< 0.001
Heart rate (bpm)	68 (13)	69 (13)	0.711
Systolic blood pressure (mmHg)	131 (190	119 (13)	0.019
Diastolic blood pressure (mmHg)	75 (11)	71 (12)	0.209
Smoking history	91 (36)	6 (32)	0.718
History of heart failure	84 (33)	0 (0)	0.003
History of coronary artery disease	48 (19)	0 (0)	0.038
Hypertension	124 (49)	0 (0)	< 0.001
Hyperlipidemia	124 (49)	0 (0)	< 0.001
Diabetes	44 (17)	0 (0)	0.047
*Function and structure*			
LV end diastolic volume (ml/m^2^)	84 (24)	75 (13)	0.090
LV end systolic volume (ml/m^2^)	43 (21)	33 (7)	0.043
LV ejection fraction (%)	51 (11)	56 (4)	0.042
LV ejection fraction < 50%	93 (36)	0 (0)	< 0.001
LV mass (g/m^2^)	61 (15)	51 (10)	0.004
RV ejection fraction (%)	53 (9)	55 (4)	0.283
*Diagnosis*			
Ischemic cardiomyopathy	29 (11)	0 (0)	
Hypertrophic cardiomyopathy	30 (12)	0 (0)	
Dilated cardiomyopathy	81 (31)	0 (0)	
Infiltrative cardiomyopathy	31 (12)	0 (0)	
Myocarditis	17 (7)	0 (0)	
Valvular disease	28 (11)	0 (0)	
Other myocardial disease^a^	43 (17)	0 (0)	
No significant findings	0 (0)	21 (100)	
*Tissue characterization*			
Late gadolinium enhancement	125 (48)	0	< 0.001
Native T1 (ms)	990 (42)	981 (28)	0.356
Post-contrast T1 (ms)	482 (39)	498 (31)	0.087
Extracellular volume (%)	27.7 (5.1)	25.9 (2.6)	0.108
*Strain*			
Global longitudinal strain (%)	− 10.5 (3.5)	− 13.5 (1.9)	< 0.001
Global circumferential strain (%)	− 12.9 (4.1)	− 16.2 (2.4)	< 0.001
Global radial strain (%)	19.6 (8.3)	25.9 (5.8)	< 0.001

Presented as mean (standard deviation) or n (%)

*LV* left ventricle, *RV* right ventricle

^a^Other myocardial disease includes regional or global left ventricular hypertrophy, non-infarct pattern of late gadolinium enhancement, and significant left ventricular or left atrial dilation

**Fig. 2:**
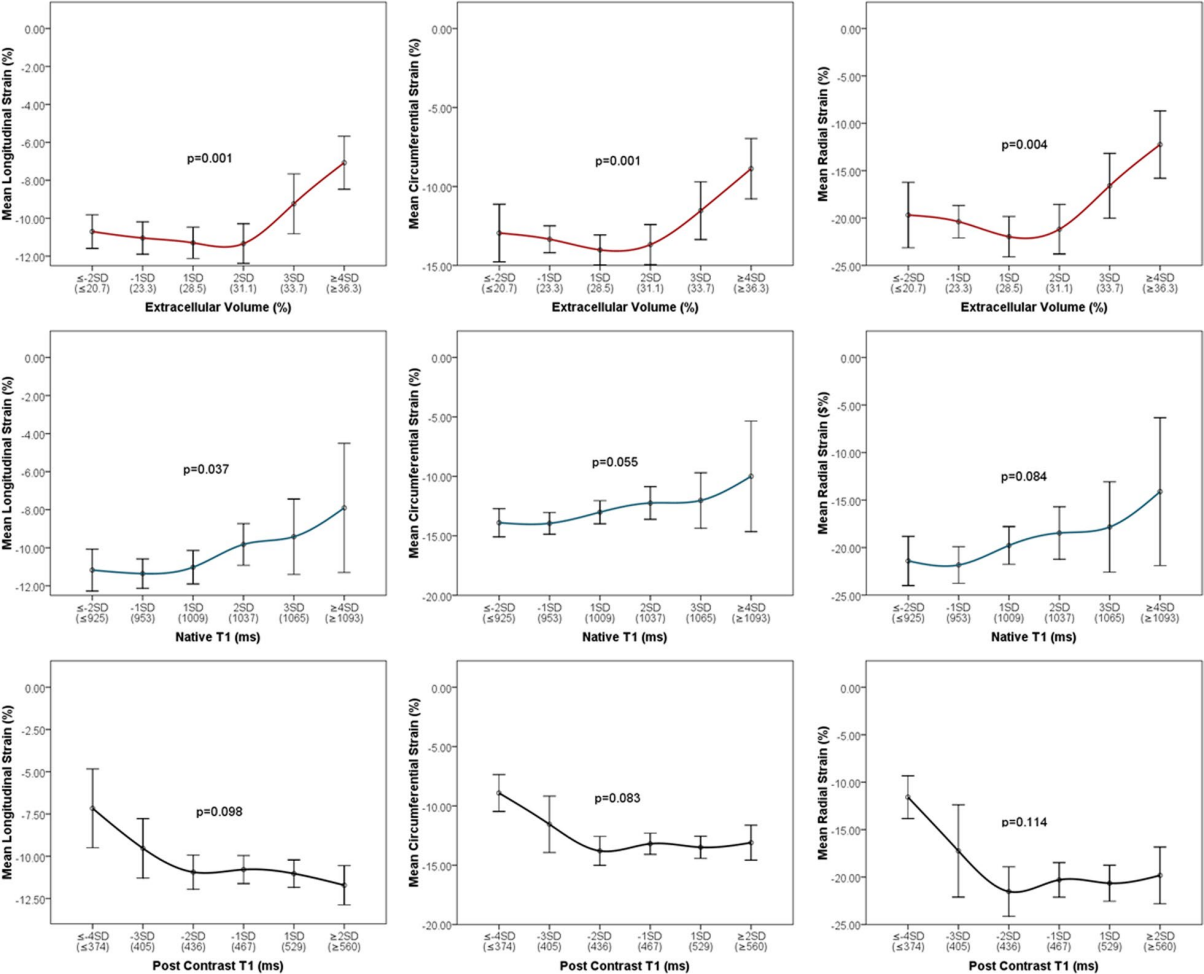
Relationships between T1 mapping parameters and global strains in all comers. The average strains and 95% confidence intervals correspond to T1 mapping parameters per standard deviation (SD) change above and below the normal mean (cut points in parenthesis). The dose–response change was tested by Jonckheere–Terpstra test for linear trend with all p < 0.05

**Fig. 3:**
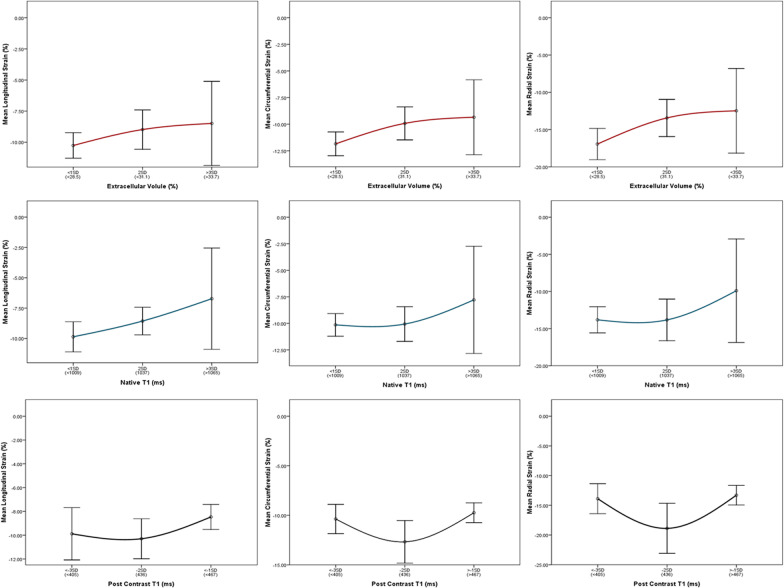
Relationships between T1 mapping parameters and global strains in a subgroup of patient with dilated cardiomyopathy (N = 81). The average strains and 95% confidence intervals correspond to T1 mapping parameters per standard deviation (SD) change above and below the normal mean (cut points in parenthesis)

The relationship between strains and the T1 parameters are shown in Fig. 2. Within the normal range of ECV (< 2SD of normal), strains of all 3 directions remained normal until ECV exceeded 2SD (31.1%) where the decrease of strain was associated with a graded increase of ECV (all p < 0.001). The decrease in strain leveled off when ECV was greater than 4SD (36.3%) and strain value became very low. Similar relationships were found between strains and native T1, where strains were reduced with a graded increase in native T1 when native T1 was greater than 1SD above the normal mean (> 1009 ms). Inverse relations were seen between strains and post-contrast T1, where strains of all 3 directions were reduced with the graded decrease of post-contrast T1 when post-contrast T1 was below 2SD of the normal mean (< 436 ms). The same comparisons were made in the largest subgroup of patients with dilated cardiomyopathy (N = 81). As expected, the T1 and ECV data range were relatively narrow compared to that in the full cohort. A similar graded relationship was present for native T1 and ECV in all strains. However, the graded inverse relationship of post-contrast T1 was present only with GLS and not with GCS or GRS (Fig. 3).

**Fig. 4:**
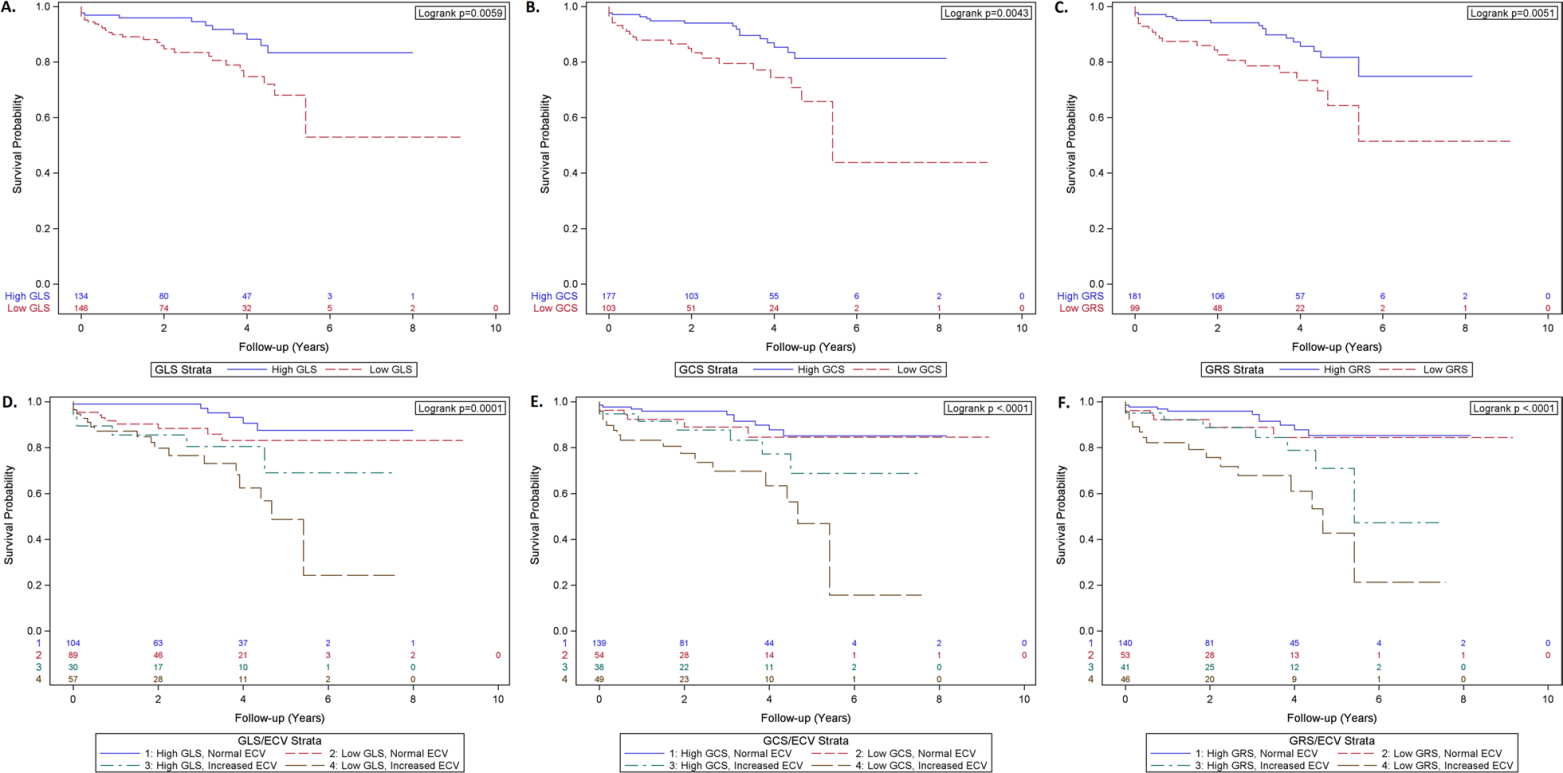
Risk of adverse clinical outcomes associated with lower global strains and higher ECV. Kaplan–Meier curves demonstrate that event-free survival is reduced with lower global strains based on the cut off (− 11.0%) for GLS (**A**), − 11.4% for GCS (**B**), and 15.7% for GRS (**C**); event-free survival is worse with increased ECV (cut off 28.3%) and reduced GLS (**D**), GCS (**E**), and GRS (**F**) using thresholds above, respectively

After a mean follow-up period of 31 ± 23 months, 41 patients reached the composite outcome, including 37 heart failure admissions and 4 deaths. Kaplan–Meier curves stratified by strain cutoff values demonstrated worsening event-free survival associated with worsening strain (Fig. 4A–C). In the subgroup analysis stratified by both strain and ECV, reduced strains were associated with reduced event-free survival predominantly in patients with increased ECV (≥ 28.3%). The worst outcome was among those with both reduced strains and increased ECV (Fig. 4D). Similar findings were seen when subgroups were stratified by strain and native or post-contrast T1 (Fig. 4E, F). Among patients with normal ECV, higher or lower strain did not seem to further discriminate survival probability. The graded worsening of outcomes in stratified analyses shown in the Kaplan-Meier plots was later confirmed by Cox proportional hazard models (Table 2).

**Table 2:** Cox proportional hazards of strain and tissue properties associated with a composite outcome of heart failure hospitalization and all-cause mortality

Primary parameter		HR	LL	UL		HR_ADJ_^a^	LL	UL
GLS ≥ − 11.0%		2.49	1.27	4.89		2.44	1.18	5.06
GCS ≥ − 11.4%		2.38	1.29	4.40		2.13	1.09	4.16
GRS ≤ 15.7%		2.34	1.26	4.33		2.23	1.16	4.27
ECV ≥ 28.3%		3.21	1.71	6.01		3.18	1.52	6.66
Native T1 ≥ 992 ms		1.62	0.88	2.99		1.51	0.78	2.94
Post-contrast T1 < 470 ms		2.25	1.21	4.21		2.02	1.01	4.03
*Hierarchical GLS and ECV model*								
GLS < − 11.0%, ECV < 28.3%		1.00	(Reference)			1.00	(Reference)	
GLS ≥ − 11.0%, ECV < 28.3%		2.41	0.89	6.52		2.24	0.82	6.12
GLS < − 11.0%, ECV ≥ 28.3%		3.65	1.18	11.32		3.05	0.87	10.64
GLS ≥ − 11.0%, ECV ≥ 28.3%		6.34	2.51	16.00		6.01	2.19	16.53

*CI* confidence interval, *GLS* global longitudinal strain, *GCS* global circumferential strain, *GRS* global radial strain, *ECV* extracellular volume, *HR*_*ADJ*_  adjusted hazard ratio

^a^Adjusted for age, gender, hypertension, diabetes, and infarct pattern on late gadolinium enhancement imaging

Cox proportional hazard models adjusting for age, gender, hypertension, diabetes and presence of infarct pattern on LGE were used to estimate the average hazard of the composite outcome over the follow-up period. Based on the dichotomized variables, reduced GLS, GCS and GRS, increased ECV, and decreased post-contrast T1 were all associated with increased hazards of the composite outcome in the adjusted and unadjusted models (Table 2). In contrast, native T1 was not independently associated with significant hazards of the composite outcome. We also tested a model including both GLS and ECV along with all other covariates which showed that reduced GLS and increased ECV were significantly associated with hazards of adverse outcomes with hazard ratios (95% confidence interval), 2.11 (1.01, 1.42) and 2.79 (1.32, 5.88), respectively.

### Reproducibility

In the reproducibility analysis, the intra-observer reliability measured by ICC was 0.992 (0.981 to 0.996) for ECV, 0.990 (0.977 to 0.996) for native T1 and 0.993 (0.985 to 0.997) for post-contrast T1. The inter-observer reliability measured by ICC was 0.961 (0.759 to 0.988) for ECV, 0.976 (0.940 to 0.990) for native T1, and 0.968 (0.854 to 0.990) for post T1. All data are presented as the averaged ICC of each component variable.

## Discussion

In this clinical cohort, we demonstrated that decreased GLS, GCS and GRS are all associated with increased ECV, increased native T1 and decreased post-contrast T1 in a dose-dependent manner. But the dose-dependent change only occurs when T1 parameters are above the normal range. Reduced strains are all associated with hazards of worsening outcomes, largely dominated by heart failure admissions. In addition, increased ECV and decreased post-contrast T1 are associated with outcome hazards but not native T1. However, the association of lower probability of heart failure free survival with reduced strain is present only when ECV is increased and not when ECV is normal. Nonetheless, those with both increased ECV and reduced strains have the worst event-free survival.

There have been investigations to uncover the tissue characteristics that lead to change in myocardial mechanical performance. Surgical specimens from a septal myectomy of patients with hypertrophic cardiomyopathy [10] and from the explanted hearts of transplant patients [11] suggest that the severity of myocardial fibrosis is an important foundation of altered longitudinal strain. While biopsy remains the gold standard in assessing myocardial fibrosis, such an invasive procedure is not feasible for a large clinical study. Therefore, a non-invasive alternative such as ECV has drawn clinical interest and is regarded as a surrogate for interstitial fibrosis [1, 2, 12]. A few small studies that investigated relationships between strain and ECV [10, 11, 13, 14] yielded mixed findings largely due to small sample size and/or narrow span of ECV distribution. A recently published large study also reported a lack of linear correlation between ECV and GLS [3]. In the present report we found that there is an important relationship between tissue features characterized by ECV, native and post-contrast T1, and strain indices including longitudinal, circumferential and radial strains. However, the relationships are not in the manner of simple linear correlations. There appears to be a distinctive deflection point above the normal mean of ECV or T1 parameters where the decrease of strains is associated with the increase of ECV or T1 in a dose–response manner. Within the normal limits of ECV or T1, strain remains largely normal. This association seems to be consistent across all 3 strain measurements. The distinctive correlative patterns of normal and abnormal ECV with strains are biologically plausible because the mechanical performance is likely to decrease when the cumulative burden of tissue abnormality crosses a critical threshold. Therefore, studies that assume a continuous linear correlation of ECV spanning the entire data range with change of strain are unlikely to establish a true relationship between the two measures. Nonetheless, the association of the 3 strain indices appear to be much stronger with ECV than with native T1 suggesting that the alteration of mechanical performance is probably more sensitive to change of ECV than to that of native T1.

At present, GLS has been widely adopted in clinical research in the investigation of myocardial mechanical performance, which is largely credited to its high reproducibility. In contrast, GCS and GRS are less reproducible by echocardiography [15]. As for CMR feature tracking both GLS and GCS are highly reproducible, but GRS has not been found to be consistently reproducible [9, 16]. It should also be recognized that modality-specific findings such as that from echocardiography or CMR are not always comparable, therefore these data should not be interchangeably interpreted [17].

Among strain indices, GLS has also been most consistently associated with risk of cardiovascular outcomes [1, 2, 4, 5]. Such observation is largely derived from studies by echocardiography; although data from CMR has recently emerged [3, 4, 18–22]. However, few reports have examined strains in all three directions in a single study. We demonstrated that not only the reduced GLS but also reduced GCS and GRS are associated with risk of adverse outcomes. In addition, event-free survival is consistently the lowest among those with reduced strains and increased ECV. The collective impact of ECV and strain highlights the intricacy and complexity of myocardial tissue and function in the pathophysiology of heart failure risk. Therefore, it is essential to assess both tissue properties and mechanical performance in order to best risk stratify patients.

### Limitations

There are several limitations to our study. First, optimal sampling for ECV calculation is debatable. We chose basal, mid and apical representative slices to aim for better coverage while some published reports favor not including the apical slice due to concerns over the partial volume effect in apical region [23]. Our approach to analyze the apical slice that is closest to the mid ventricle allowed us to avoid the thin apical wall and ensure good reproducibility. While ECV assessment based on 3 slices generated better coverage for tissue characterization than that of 2 slices, it may still underestimate the disease burden particularly for disease with heterogeneous distribution of fibrosis. The future development of 3D T1 mapping with free breathing will improve coverage but will not burden the patient. Second, our cohort was from a single center with a relatively modest population. However, we were able to demonstrate the intricate link between tissue property alteration and mechanical impairment, as well as their collective impact on adverse clinical outcomes. Third, this was a clinically referred cohort with mixed diagnosis of ischemic and non-ischemic diseases. While a particular referring pattern may be subjective to institutional practice, the collective cases in our cohort have typical representations of common CMR referrals, thereby making our findings generalizable. Fourth, the absolute value of GLS appears to be low, which is subject to the selection of feature tracking software as we demonstrated in our previous publication [9]. We used feature tracking for strain analysis in our study because it has the advantage of using cine images that are routinely captured nearly in all clinical cases. But we acknowledge other important modalities for strain evaluation such as tagged cine images, SENC and displacement encoding with stimulated echoes (DENSE) which allow for myocardial deformation to be analyzed using features encoded directly onto the myocardium [24–26] although additional dedicated breath-hold imaging is required that adds scan time and burden to the patient. Finally, this is a single center study largely limited to patients of Caucasian background. The incidence of cardiac death was low. In addition, the outcome event rate was low in the subgroup of dilated cardiomyopathy (12 out of 81) rendering insufficient statistical power for outcome analysis. Future studies with multi-center designs and large clinical cohorts are warranted to compare ECV and strain with other well-established biomarkers, such as B-type natriuretic peptide and diastolic function, to improve risk stratification.

## Conclusion

There is a dose–response relationship between tissue abnormalities assessed by T1 parameters and impairment  of GLS, GCS and GRS. Reduced strains are associated with reduced event-free survival predominantly in patients with increased ECV. Those with both increased ECV and reduced strains have the worst outcome. Our findings highlight the intrinsic link between the tissue property alteration and the myocardial mechanical impairment in the pathophysiology of heart failure and additionally demonstrate an essential role of tissue characterization in risk stratification among patients with reduced strain.

## Data Availability

The datasets generated and/or analyzed during the current study are not publicly available due the need to maintain patient privacy/confidentiality but are available from the corresponding author on reasonable request.

## Abbreviations

CMR: Cardiovascular magnetic resonance
DENSE: Displacement encoding with stimulated echoes
ECG: Electrocardiogram
ECV: Extracellular volume
GCS: Global circumferential strain
GLS: Global longitudinal strain
GRS: Global radial strain
LGE: Late gadolinium enhancement
LV: Left ventricle/left ventricular
LVEF: Left ventricular ejection fraction
PSIR: Phase sensitive inversion recovery
RV: Right ventricle/right ventricular
