# Correction to: The association of reduced left ventricular strains with increased extracellular volume and their collective impact on clinical outcomes

**DOI:** 10.1186/s12968-021-00826-0

**Published:** 2021-11-18

**Authors:** Chunna Jin, Jonathan Weber, Harsimar Singh, Kathleen Gliganic, J. Jane Cao

**Affiliations:** 1 St Francis Hospital & Heart Center, 100 Port Washington Blvd, Roslyn, NY 11576 USA; 2 State University of New York at Stony Brook, 100 Nicholls Road, Stony Brook, NY 11794 USA; 3 Department of Cardiology, Second Afliated Hospital of Zhejiang University, Hangzhou, 310009 Zhejiang China

## Correction to: J Cardiovasc Magn Reson (2021) 23:93 10.1186/s12968-021-00776-7

After publication of the original article [1] the authors found a minor error in the article:

- Figures 2 and 3 of the article incorrectly labeled Mean Radial Strain (%) as a negative value instead of positive. This affects the displayed scale of radial strain on the y-axis of plots within both figures. In the initial submission we presented the radial strain in a positive scale. In order to keep the presentation consistent with longitudinal and circumferential strains we decided to adjust the display scale of radial strain in the final submission which we believe is helpful for the reader to interpret the findings. Unfortunately, the negative scale on y-axis was carried out for all three strains. After careful examination of the data used to create the plots, we concluded that the error lies with the y-axis label alone and not the data nor relationships among variables displayed in Figs. 2 and 3. This minor error should not affect the interpretation of results or implications of our work.

The original article has been updated to amend the errors in Figs. 2 and 3. The incorrect and correct figures are shown in this erratum (Figs. 1, 2, 3 and 4).

**Fig. 1:**
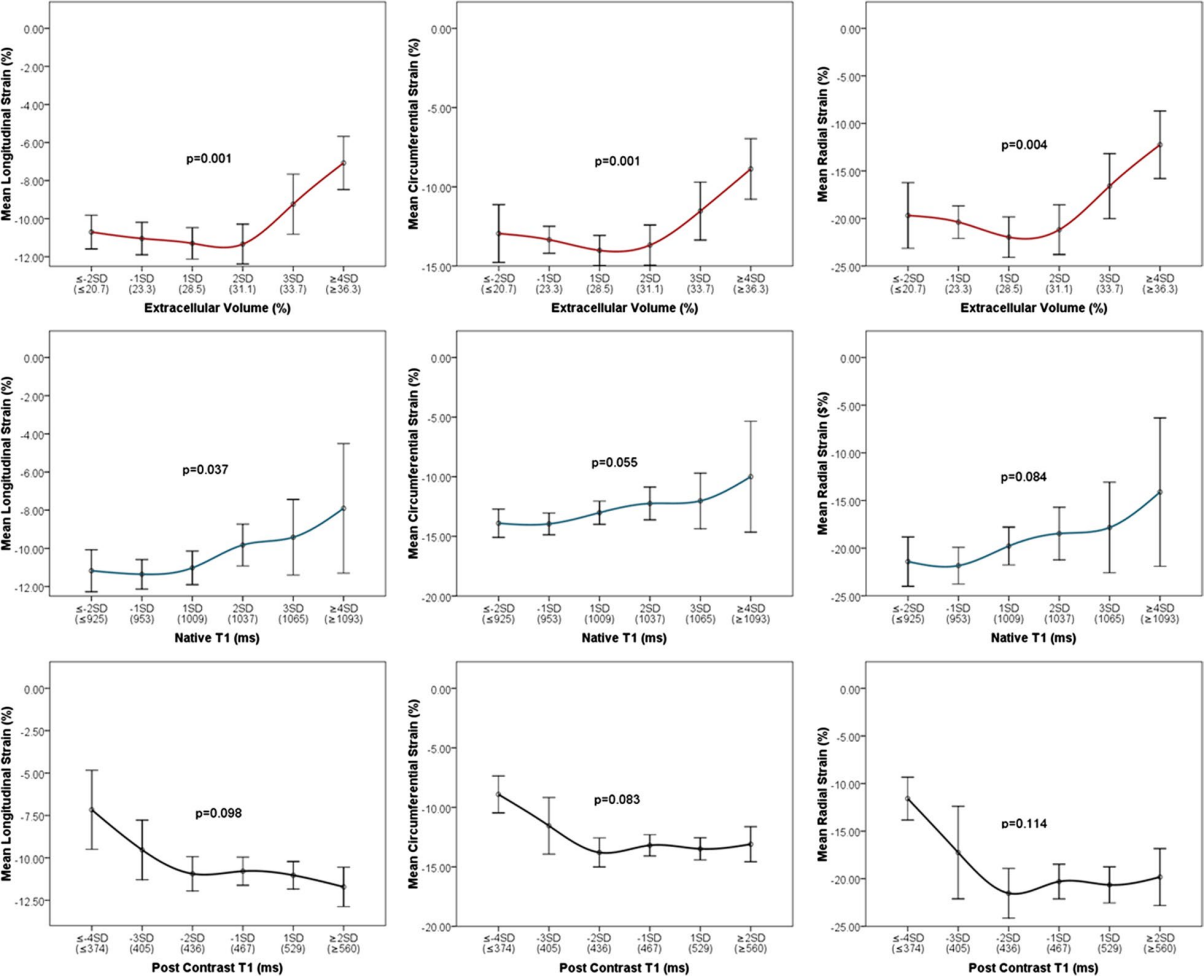
Incorrect version of Fig. 2

**Fig. 2:**
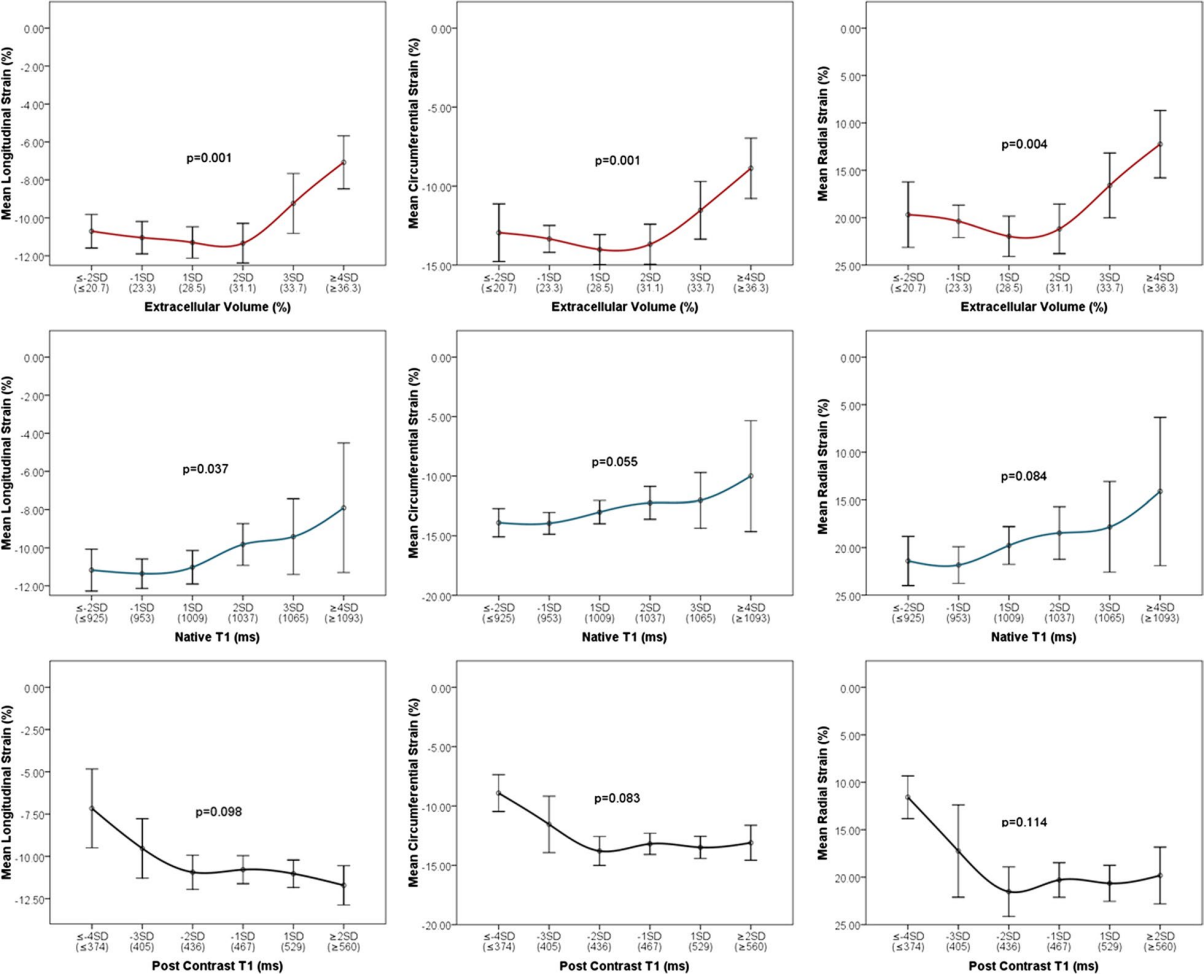
Correct version of Fig. 2

**Fig. 3:**
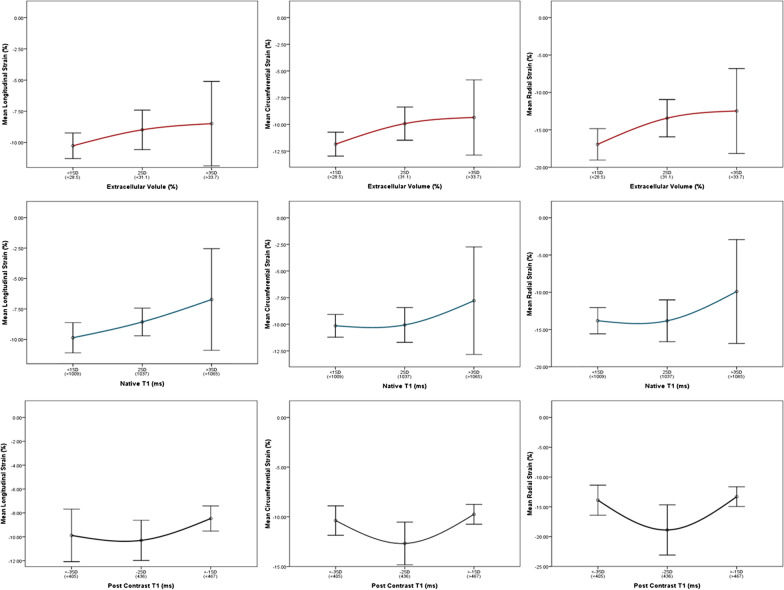
Incorrect version of Fig. 3

**Fig. 4:**
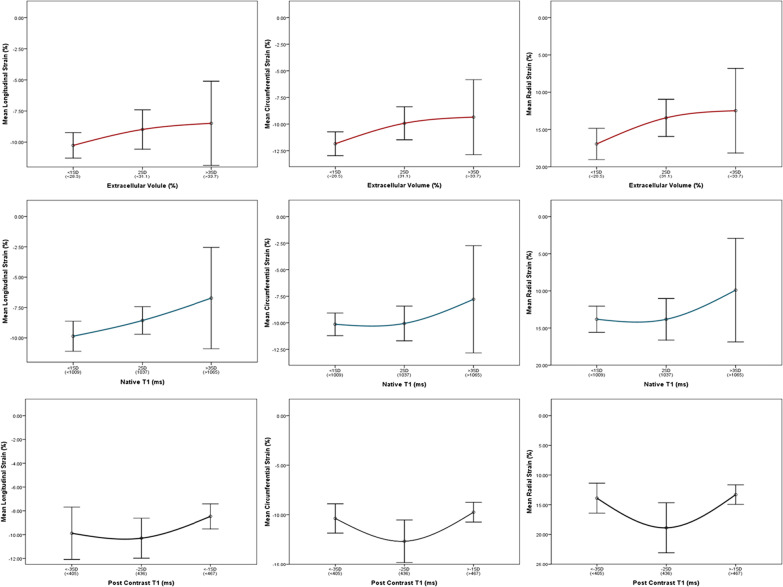
Correct version of Fig. 3
